# Public Health Surveillance in Electronic Health Records: Lessons From PCORnet

**DOI:** 10.5888/pcd21.230417

**Published:** 2024-07-11

**Authors:** Nidhi Ghildayal, Kshema Nagavedu, Jennifer L. Wiltz, Soowoo Back, Tegan K. Boehmer, Christine Draper, Adi V. Gundlapalli, Casie Horgan, Keith A. Marsolo, Nik R. Mazumder, Juliane Reynolds, Matthew Ritchey, Sharon Saydah, Yacob G. Tedla, Thomas W. Carton, Jason P. Block

**Affiliations:** 1 Department of Population Medicine, Harvard Medical School, Harvard Pilgrim Health Care Institute, Boston, Massachusetts; 2 National Center for Chronic Disease Prevention and Health Promotion, Centers for Disease Control and Prevention, Atlanta, Georgia; 3 Office of Public Health Data, Surveillance, and Technology, Centers for Disease Control and Prevention, Atlanta, Georgia; 4 Department of Population Health Sciences, Duke Clinical Research Institute, Duke University School of Medicine, Durham, North Carolina; 5 Department of Internal Medicine, University of Michigan Health, Ann Arbor, Michigan; 6 Coronavirus and Other Respiratory Viruses Division, Centers for Disease Control and Prevention, Atlanta, Georgia; 7 Department of Medicine, Vanderbilt University Medical Center, Nashville, Tennessee; 8 Louisiana Public Health Institute, New Orleans, Louisiana

## Abstract

**Introduction:**

PCORnet, the National Patient-Centered Clinical Research Network, is a large research network of health systems that map clinical data to a standardized data model. In 2018, we expanded existing infrastructure to facilitate use for public health surveillance. We describe benefits and challenges of using PCORnet for surveillance and describe case studies.

**Methods:**

In 2018, infrastructure enhancements included addition of a table to store patients’ residential zip codes and expansion of a modular program to generate population health statistics across conditions. Chronic disease surveillance case studies conducted in 2019 assessed atrial fibrillation (AF) and cirrhosis. In April 2020, PCORnet established an infrastructure to support COVID-19 surveillance with institutions frequently updating their electronic health record data.

**Results:**

By August 2023, 53 PCORnet sites (84%) had a 5-digit zip code available on at least 95% of their patient populations. Among 148,223 newly diagnosed AF patients eligible for oral anticoagulant (OAC) therapy, 43.3% were on any OAC (17.8% warfarin, 28.5% any novel oral anticoagulant) within a year of the AF diagnosis. Among 60,268 patients with cirrhosis (2015–2019), common documented etiologies included unknown (48%), hepatitis C infection (23%), and alcohol use (22%). During October 2022 through December 2023, across 34 institutions, the proportion of COVID-19 patients who were cared for in the inpatient setting was 9.1% among 887,051 adults aged 20 years or older and 6.0% among 139,148 children younger than 20 years.

**Conclusions:**

PCORnet provides important data that may augment traditional public health surveillance programs across diverse conditions. PCORnet affords longitudinal population health assessments among large catchments of the population with clinical, treatment, and geographic information, with capabilities to deliver rapid information needed during public health emergencies.

**Summary**

**What is already known on this topic?**

Existing survey-based surveillance programs provide important information on the epidemiology of chronic and infectious diseases. Electronic health record (EHR) data can be used to supplement surveillance efforts.

**What is added by this report?**

In this study, we describe the attributes and challenges of using EHR data for disease surveillance. We describe surveillance case studies and future directions for enhancing opportunities to use EHR data for public health surveillance.

**What are the implications for public health practice?**

EHR data have an important role for public health surveillance both for chronic and infectious diseases, providing comprehensive information available soon after data collection. Strategic funding and financing models need to be developed, and federal, state, and local support could help establish EHRs as an important sustainable mechanism for surveillance.

## Introduction

Electronic health records (EHRs) contain extensive longitudinal health information about patients and populations (1). Over the last decade, prompted by federal meaningful use guidelines and incentives, EHRs have become ubiquitous in health care settings (2). Because of their wide availability, EHRs are a viable option for disease surveillance and have some advantages over traditional survey-based surveillance methods, such as the National Health and Nutrition Examination Survey and the Behavioral Risk Factor Surveillance System (Table 1) (3,4).

**Table 1:** Surveillance System Attributes for Traditional Sources of Surveillance Information and Electronic Health Records (EHRs)

Surveillance system attributes	Traditional national surveillance surveys^a^		EHRs^b^	
	Strengths	Weaknesses	Strengths	Weaknesses
**Timeliness**	NA	Can take years between data collection and availability	Available soon after collected	NA
**Content and scope**	In-depth availability of patient-reported data on behaviors; extensive collection of social determinants of health data	Limited sample sizes, especially for less common sociodemographic groups	Data on millions of patients provides ability to estimate disease prevalence for rare diseases, less common subgroups (Native Hawaiian/Pacific Islander, American Indian/Alaska Native), and small area geographic units and population-based cohorts	Limited availability of patient-reported data; social determinants data availability increasing but limited to insurance type and linked Census data for many EHRs
**Structured data; data subjectivity; longitudinal data**	Objectively measured health outcomes (vitals, laboratory values) according to study protocol	Cross-sectional or panel designs limit longitudinal follow-up	Longitudinal follow-up on patients allows tracking changes over time; data available on disease control over time	Many data are unstructured (eg, patient notes) and less available for use; structured data standardization is variable; identification of diseases often depends on use of nonspecific diagnostic codes; prescription data typically available but pharmacy dispensing may not be
**Representativeness**	Nationally representative by design; typically covers entire US population with probability-based sampling strategies	Certain populations can be under-represented (eg, people without a landline telephone, the institutionalized population); characteristics of respondents may differ from nonrespondents in measured or unmeasured ways	Some research networks have data available on people in all US states and territories; patients with multiple types of insurance (commercial and government insurance) are typically available	Representative of care-seeking population, which may limit broad surveillance questions at the population level; representativeness of urban versus rural populations dependent on institutions contributing data
**Data quality, completeness**	Data collected according to study protocol; robust data completeness and curation	Telephone surveys used in some programs reliant on self-report; all surveys subject to nonresponse	Objective measures of some disease (eg, diabetes, obesity) and robust computable phenotypes of others	Missing data are common; data not collected according to a standardized protocol
**Resources required**	Infrastructure established by federal agencies to collect data; sampling and weighting strategies well validated and centrally applied by data collectors; some flexibility on adding new questions and data elements	Requires substantial resources and staff to facilitate	Data collected for routine clinical activities and only additional resources for collection required for new data elements	Data processing requires substantial resources, especially to address data quality issues that can arise; adding new data elements challenging

Abbreviation: NA, not applicable.

a Examples: National Health and Nutrition Examination Survey (NHANES, www.cdc.gov/nchs/nhanes), Behavioral Risk Factor Surveillance System (BRFSS, www.cdc.gov/brfss).

b Example: National Patient-Centered Clinical Research Network (PCORnet).

Some of the most important attributes of EHRs for surveillance include timeliness of data and availability for large populations. EHR data are collected daily through routine clinical care delivery and can be made available quickly if resources are available for processing and data curation. In contrast, large national surveillance programs typically use surveys or field data collection, followed by data processing that can lead to extensive lag times between data collection and availability. The scope of EHR data available also can provide important granular information about subgroups. For example, although retrieving metro area and small area modeling estimates via national surveillance surveys is possible, these data are often restricted for privacy reasons and, in some cases, are imputed rather than directly measured (4–6). Furthermore, the sample size of surveys limits the availability of data on rare conditions or less common subgroups of individuals, such as among racial and ethnic minority groups (4,6). Because of the availability of data on vast populations that allow for numbers large enough to stratify by even uncommon subgroups, EHRs can provide data for specific geographic regions and populations (3,6,7).

Another area of potential benefit of EHRs for surveillance is the availability of longitudinal objective, measured data, such as vital signs and laboratory values. These data allow for more accurate definitions (ie, phenotypes) of disease, such as using a combination of medication prescriptions, laboratory values, and vital signs to define chronic disease (4). Measured data can also enable an objective determination of disease severity and disease control over time, such as defining whether patients are meeting guideline control targets for diabetes or hypertension by using glycosylated hemoglobin or measured blood pressure values. These data can provide information both cross-sectionally and longitudinally in cohorts that receive care over time. National surveillance surveys typically rely on self-reported information or single vital sign or laboratory values to define disease prevalence and incidence. Lastly, EHRs can offer longitudinal information with short latency, allowing for capture of information on changing health status, in contrast to that collected through the lengthy process of repeated survey administration (7).

EHR data present some challenges, including with data quality and representativeness. Missing data also are common for myriad reasons. The fragmented health care system in the US precludes comprehensive data integration across care settings, and patients often receive care in multiple institutions with different data systems (8,9). Even when information from other health care institutions can be viewed within the health care system that serves as the medical home for a patient (eg, *Care Everywhere* in Epic), that information may not be captured in clinical data warehouses that can be used for surveillance. Clinical notes written in free text may not be easily translated to structured data fields, resulting in missing information on symptoms and exposures (10). Furthermore, clinical data available in EHRs do not typically include information on social determinants, quality-of-life measures, and other health behavior information that could be more readily collected through national surveys. Some social determinants data can be integrated when available geographic information can be linked to community-level data from the US Census and other data resources (11,12). Another drawback of EHR surveillance is that data may not be representative, and clinical practice patterns may differ between sites, leading to heterogeneity in data available due solely to the differential ascertainment of diagnoses, for example. Exploration of the epidemiology of disease by geography also is heavily dependent on the number of institutions per geographic area providing data. EHR data have information on patients who are seeking care, likely biasing inferences toward certain demographic groups receiving more medical care and patients who have chronic conditions, have health insurance coverage, or live in urban areas (12). However, unlike claims data that are typically limited to commercial insurance, Medicaid, or Medicare separately, EHR data are typically agnostic to payer source and have information on patients with a diverse array of insurance sources, including those who are uninsured (13) (Table 1).

In this article, we discuss the use of EHR data for public health surveillance in a large national research network and present case studies of its use for chronic disease and its later adaptation for COVID-19 surveillance during a public health emergency.

## Methods

### PCORnet as a data source for public health surveillance

PCORnet, the National Patient-Centered Clinical Research Network, is a research infrastructure program that was established to support use of health care data for comparative effectiveness research (14). This network-of-networks includes more than 60 health care systems embedded in 8 Clinical Research Networks (https://pcornet.org/network/), with a regulatory infrastructure that prioritizes data sharing while protecting patient privacy. Data from millions of patients from different source EHR systems are harmonized locally into a standard data set, called a Common Data Model (CDM). This CDM is updated over time to incorporate new and evolving data elements and is nearly identical across all participating institutions, allowing for centralized querying and interoperability of data across sites (15). The data elements include comprehensive clinical information, including prescriptions, diagnoses, procedures, vital measures, laboratory values, and geographic information, among other data elements, from all care settings relevant for a specific health care system (eg, ambulatory, emergency department, inpatient).

Quarterly data quality reviews allow for a comprehensive assessment of conformance, completeness, plausibility, and persistence, with feedback provided on issues discovered. Data sharing across the network is accommodated by a Master Data Sharing Agreement, with further regulatory processes outlined to accommodate varied circumstances required for research and operations (14,16). PCORnet also has a “front door” mechanism for investigators to request data queries or study collaborators (17).

PCORnet has a distributed query infrastructure, and users can submit a query and obtain a coordinated response that combines data across participating health systems (16). PCORnet also is an engaged network in which investigators, informatics specialists, clinicians, patients, and other partners from sites can provide context and information regarding the data available from that site.

Reusable SAS-based tools that have been developed for PCORnet are available for querying data, with regular updates for CDM changes and to enhance functionality for new data needs. These tools are modular descriptive programs that can be quickly adapted to create and characterize cohorts with aggregate data, using tables and variables defined in the PCORnet CDM. While PCORnet has protocols allowing for the transfer of patient-level data to requestors, the availability of a reusable process for obtaining aggregate data from partners allows for assessments that can often be completed quickly. While less flexible than centralized, pooled data available for analysis, aggregate data are typically sufficient for surveillance.

PCORnet has several capabilities that foster successful public health surveillance. As a national EHR surveillance program with multiple contributing entities, PCORnet contains data on more than 30 million patients annually (16). PCORnet has broad geographic representation with most sites providing data from both inpatient and outpatient settings (https://pcornet.org/data/). The network provides access to patients with longitudinal follow-up, often over many years; populations large enough to allow for examination of subgroups, such as by race and ethnicity, geography, and multimorbidity; and opportunities to capture adequate numbers of patients with rare diseases to make important inferences about prevalence. Data captured on race are considerably more complete than those found in some other commonly used clinical data sets (18). For example, among all patients with encounters in 34 PCORnet sites during October 2022 through December 2023, race and ethnicity information was missing for 9.5% of those younger than 20 years and 8.7% of those aged 20 years or older (Table 2). Race and ethnicity missingness was lower for patients with diagnostic codes for COVID-19, positive laboratory tests for SARS-CoV-2, or recent prescriptions for COVID-19 medications: 7.6% for patients younger than 20 years and 5.1% for patients aged 20 years or older (data not shown).

**Table 2:** Racial and Ethnic Characteristics of Patients in 34 PCORnet Sites, October 2022 Through December 2023

Race and ethnicity	Children, adolescents, young adults (aged <20 y)	Adults (aged ≥20 y)
	N (%)	
NH American Indian/Alaska Native	32,351 (0.4)	100,070 (0.5)
NH Asian	276,545 (3.4)	686,624 (3.2)
NH Black or African American	1,266,244 (15.7)	2,973,069 (13.8)
Hispanic^a^	1,743,201 (21.6)	3,246,099 (15.1)
NH Multiple race	115,798 (1.4)	54,400 (0.3)
NH Native Hawaiian/Other Pacific Islander	23,348 (0.3)	40,290 (0.2)
NH Other race	233,508 (2.9)	436,754 (2.0)
NH White	3,746,223 (46.5)	12,430,453 (57.8)
Missing	768,425 (9.5)	1,869,061 (8.7)

Abbreviations: NH, Non-Hispanic; PCORnet, National Patient-Centered Clinical Research Network.

a Includes any patients with a designated ethnicity as Hispanic, regardless of race. All racial groups had ethnicity categories of non-Hispanic or missing/other Hispanic ethnicity.

In 2018, PCORnet began to expand capabilities of the network to conduct EHR-based surveillance, specifically focused on chronic disease. The program was initially used for pilot projects that built capacity for geographic data capture. In March 2020, the network began exploring whether its resources, including the newly established capabilities for chronic disease surveillance, could be adapted for COVID-19 surveillance. This shift required some changes, especially to provide more timely data. PCORnet expanded its infrastructure to include the ability to frequently, up to twice monthly, refresh data. With regularly refreshed data and modular programs, data can now be available for public health professionals and researchers in a matter of weeks. Simple analyses that only require basic counts and frequencies can be provided even more quickly.

### Expanded data and tools for surveillance and case studies in PCORnet

Starting in 2019 with CDM version 5.0, PCORnet incorporated a new, optional CDM table containing patient-level geographic information. This table allowed sites to include patient information on 9- and 5-digit zip code, city, state, and the start and end date for that address information. To accommodate surveillance queries in PCORnet, we developed a geographic assessment module to query this address data (16). The module allows for the characterization of a cohort based on the most recent address stratified by zip code, city, state, or Census region. Queries also can pull patient-level data with zip-code or mapped US Census Bureau’s data elements. The geographic module was piloted at several PCORnet health systems for chronic disease surveillance case studies, including atrial fibrillation (AF) and liver cirrhosis.

Starting in April 2020, select PCORnet institutions collaborated on a response to the COVID-19 pandemic that would allow for more frequent querying of data. Institutions developed a CDM that contained data for a subset of their total patient population, including only patients who had a diagnostic code for a respiratory virus or infection or a viral laboratory test for SARS-CoV-2. The inclusion criteria for this subset CDM were later expanded to include COVID-19 therapeutics and vaccines. Filtering the broader population using these criteria allowed for quicker refreshes of data, facilitating reports on data with a latency of a few weeks, in contrast to the regular quarterly updates. This process also was a more practical approach for sites, given that frequent refreshes of their complete patient population data would take extensive effort and data storage. Sites initially updated their filtered CDM biweekly and then later monthly or on request.

The PCORnet team leading this surveillance effort also changed the modular statistical programs to allow for characterization of cohorts using results of qualitative viral testing information, available mortality information (ie, typically deaths reported to the health care system or in-hospital deaths) and records of vaccinations given in the health care system or populated in EHRs from state registry linkages, when available. The statistical programs also were updated to allow for distributed advanced analytics, including the use of multiple regression models that execute behind institutions’ firewalls and return only summary model output; these results can be combined across sites using meta-analytic techniques (19). Since October 2020, PCORnet has participated in a cooperative agreement funded by the Centers for Disease Control and Prevention (CDC) to provide COVID-19 information from up to 43 PCORnet institutions on a biweekly basis.

## Results

The geographic query module was released for use in PCORnet in July 2019. Geographic data returned from queries were well distributed but contained many sparsely populated zip codes. Zip code data typically were not available retrospectively; many sites only began capturing the geographic information prospectively at the time of its CDM release. County information was added to the CDM during the release of CDM 6.1 in April 2023.

By August 2023, 62 of 63 (98%) PCORnet institutions had populated geographic information. Among sites, 59 (94%) had at least some information on 5-digit zip code, with 53 (84%) having 5-digit zip available on at least 95% of their patient population. For 9-digit zip codes, 42 (67%) sites had some information on patients, with 10 (16%) having this information populated for at least 75% of their population. The pilot projects on AF and liver cirrhosis tested the implementation of this geographic data table and use of the geographic query module.

### Oral anticoagulant use

Oral anticoagulant (OAC) therapy is proven to reduce the risk of stroke and is the standard treatment for stroke risk reduction in patients with AF (20,21). Some local studies have found that about half of patients with AF at risk of stroke do not get OAC prescriptions (22–25). However, little information exists on the rate of prescriptions of OACs across US states. We used data from 4 PCORnet Clinical Research Networks (CAPriCORN, STAR, REACHnet, and ADVANCE) and investigated the OAC prescription rate in 22 states. Patients newly diagnosed with AF between January 2014 and December 2019, with a CHADSVASC score of 2 or more, no history of stroke, and known zip code were included in our analysis. The CHADSVASC score includes information on risk factors for stroke among patients with AF and is used to calculate a predicted probability of stroke; a score of 2 or more is considered high risk for stroke (26). Among 148,223 newly diagnosed AF patients eligible for an OAC, 43.3% were on any OAC, 17.8% received any warfarin, and 28.5% received any novel oral anticoagulant (NOAC) in the year following AF diagnosis. OAC prescription rates varied greatly across states, ranging from 28.4% in Virginia to 54.0% in Indiana.

OAC prescriptions continue to be low in patients with AF and vary across health systems and geographic regions. These results are consistent with findings from previous studies (22–25). Our findings provided comprehensive information on OAC use across regions but were not nationally representative. The study only examined health systems that were part of the CRNs involved in the study: 6 from CAPriCORN, 2 from REACHnet, and 1 each from STAR and ADVANCE.

### Cirrhosis

Cirrhosis, irreversible damage to the liver, is a leading cause of illness and death in the US (27). Despite its importance as a major medical condition, one of the most important challenges for determining population prevalence and geographic distribution is the lack of a unified repository of patients with cirrhosis. PCORnet provided an opportunity to explore the epidemiology of cirrhosis using diagnostic codes in EHRs. In this pilot study, we included any patient aged 18 years or older with a qualifying *International Classification of Diseases* (ICD) code for cirrhosis (ie, ICD-9 or ICD-10) who received care at a participating center during the calendar years 2015–2018. The study included 9 health systems from 3 Clinical Research Networks, with strong overlap with the AF pilot: STAR, CAPriCORN, and REACHnet. Patient zip code was assessed as zip code of residence both within 90 days of cohort inclusion and within any prior period before inclusion.

Overall, we identified 60,268 patients with ICD codes for cirrhosis. Patients were 58% (n = 34,908) male, 57% (n = 34,458) White race, and 81% (n = 48,646) non-Hispanic ethnicity, with a mean age of 58 years. The most common etiologies for cirrhosis were hepatitis C (n = 13,882; 23%) and alcohol (n = 13,187; 22%); however, nearly half of patients (n = 29,177, 48%) did not have a clear etiology of liver disease documented in the EHR. When geographic data were restricted to a period that was within 90 days of diagnosis of cirrhosis, residential zip code was highly missing (86% missing); missingness was much lower for records of any zip code documented in the EHR before study inclusion (33% missing). This study was conducted relatively soon after the geographic information was first provided in the CDM. Because most sites populated their geographic information prospectively, missingness will improve over time, allowing for PCORnet to be effectively used for important public health surveillance of cirrhosis by geography.

### COVID-19 surveillance

PCORnet was able to quickly transition to infectious disease surveillance and began reporting COVID-19 national data in April 2020. Since October 1, 2020, working with CDC, 43 PCORnet institutions have been engaged in a broad surveillance effort in which queries are conducted up to twice monthly on varied topics, with aggregate data provided to CDC in support of pandemic response. The surveillance effort has led to over 50 data queries. In a recent query, focused on the period of October 1, 2022, to December 31, 2023, 34 of the participating PCORnet institutions recorded 887,051 patients aged 20 years or older and 139,148 patients younger than 20 years who tested positive for SARS-COV-2, received a COVID-19 therapeutic, or had an ICD-10 code for COVID-19, with geographic information available by state. Among these patients, 80,712 (9%) of the patients aged 20 years or older and 8,322 (6%) of the patients younger than 20 years were cared for in the inpatient setting. We have mapped zip code data available in this population to designated geographic variables, including US Census Bureau variables for rurality and urbanicity and area deprivation index. These variables were well populated with missing data for 3%–4% of the population. Most patients lived in urban settings (88%–89%). For area deprivation index scores, 44% of patients aged 20 years or older and 51% of patients younger than 20 years were in the top 2 quartiles (ie, higher area deprivation).

This COVID-19 surveillance program has generated important information on the prevalence of post-acute sequelae of SARS-CoV-2 infection (28), disparities in uptake of COVID-19 therapeutics (18,29), cardiac complications after COVID-19 mRNA vaccines and SARS-CoV-2 infection (30), and association of uncontrolled diabetes and hypertension and severe COVID-19 (19). Information also was captured on trends in chronic and infectious disease incidence and preventive care services before and during the pandemic and the incidence of and therapeutics for mpox to support CDC’s response. The infrastructure developed for this CDC-funded project also was leveraged for other large-scale research programs, such as providing preliminary data for the National Institutes of Health RECOVER Initiative (31).

## Discussion

With the availability of patient-level geographic information, large populations, and comprehensive longitudinal clinical data, PCORnet and similar networks can fill in gaps for existing national surveillance infrastructure. Pilot surveillance projects provided valuable lessons for use of PCORnet infrastructure that was leveraged for the national COVID-19 public health response.

Streamlined regulatory processes are critical to accommodate efficient surveillance work. For example, we pursued individual institutional review board (IRB) approvals for each chronic disease surveillance pilot project; all IRB approvals required more than 6 months to complete. Lead sites faced difficulties in coordinating single IRBs (eg, through SMART IRB) across participating sites and ascertaining whether sites should be obtaining IRB determination for limited or nonlimited data sets. These processes were streamlined during the COVID-19 pandemic. The collaborative PCORnet CDC COVID-19 project was exempt from IRB review because it constituted public health surveillance required or authorized by a public health authority, as specified under the Common Rule. Our experience demonstrates that clear network guidance on approvals necessary for varied types of data exchange could help streamline surveillance projects. Fewer requirements should be needed for projects using only aggregate data, even with the inclusion of some geographic information (32). This guidance will be most important for surveillance projects that are not directed by a public health authority and, thus, not exempt from IRB review (33).

Implementation of the pilot projects also revealed both issues and benefits that arose from using various organizing units for geographic data. The city variable was not useful due to varied spellings. Zip code was well-populated at the 5-digit level, providing expanded geographic capabilities that go beyond other data sources, such as insurance claims. Counties can be useful in some cases, such as for states that use geographic divisions other than standard ones (eg, Utah Small Area Codes); county was added as a new geographic unit for the PCORnet CDM in 2023. Ultimately, census tracts or block groups are likely most useful because these geographic units are typically more uniform than larger zip code areas. Regulatory processes could help ease the way for networks to use this information more readily. Direct linkage to US Census Bureau and other community-level data might also preclude the need to share actual geographic identifiers.

### Implications for practice

EHR-based networks have important potential for surveillance of key priority areas that align with health and public health missions. When rapidly refreshed data with short latency are required, PCORnet has shown that it can be used for COVID-19 surveillance and other infectious disease outbreaks or epidemics, with data that are available with often very short latency from the time of collection. These rapidly available data in PCORnet allowed for timely reporting of infection trends, including information on patient demographics, comorbidities, and treatments used or prescribed. Timely data can also be important for chronic disease surveillance but may not require updates as frequently as for emerging infectious diseases.

EHR data could be improved to address some of the challenges for its use in surveillance. Developing and deploying population statistical weighting schemes for data in EHRs, which have data only on patients seeking care from designated health care institutions, could help alleviate issues related to generalizability of populations (34,35). Strategic funding and financing models should be developed, and federal, state, and local support could help establish EHRs as an important sustainable mechanism for surveillance (36). The continued success of using PCORnet for large-scale surveillance also can expand its engagement of partners to ensure that data can be used most efficiently to support population health priorities (36).
